# Occurrence of Virulence Genes among Methicillin-Resistant *Staphylococcus aureus* Isolated from Subclinical Bovine Mastitis

**DOI:** 10.1021/acsomega.3c04206

**Published:** 2023-10-02

**Authors:** Maryam Shahid, Riaz Hussain, Zeeshan Nawaz, Bilal Aslam, Muhammad Zishan Ahmad, Abu Baker Siddique, Hira Ahsan, Aiman Fatima, Iahtasham Khan, Bilal Mustafa, Rashid Iqbal, Khalid M. Al Syaad, Ashwag Shami

**Affiliations:** † Institute of Microbiology, Government College University, Faisalabad 38040, Pakistan; ‡ Department of Pathology, Faculty of Veterinary and Animal Sciences, The Islamia University of Bahawalpur, Bahawalpur 63100, Pakistan; § Department Veterinary Pathology, Faculty of Veterinary and Animal Sciences, PMAS Arid Agriculture University Rawalpindi, Rawalpindi 46000, Pakistan; ∥ Institute of Microbiology and Molecular Genetics University of the Punjab Lahore, Lahore 54590 Pakistan; ⊥ Section of Epidemiology and Public Health, Department of Clinical Sciences, University of Veterinary and Animal Sciences, Lahore Sub-Campus, Jhang 35200, Pakistan; # University of Oxford, Department of Biology, Wildlife Conservation Research Unit (WildCRU), Oxford Ox13 5QL, U.K.; ¶ Department of Agronomy, Faculty of Agriculture and Environment, The Islamia University of Bahawalpur Pakistan, Bahawalpur 63100, Pakistan; ∇ Biology Department, College of Science, King Khalid University, Abha 61413, Saudi Arabia; ○ Department of Biology, College of Science, Princess Nourah bint Abdulrahman University, Riyadh 11671, Saudi Arabia

## Abstract

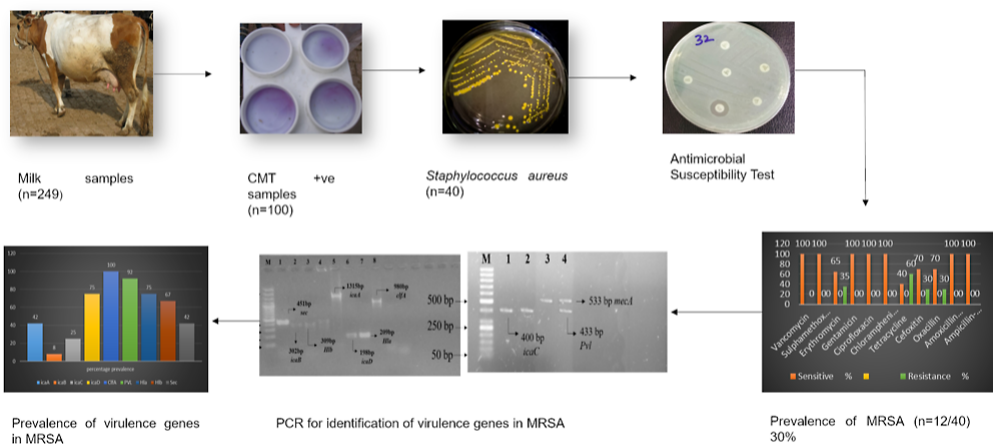

The occurrence of *Staphylococcus aureus*-induced subclinical mastitis holds significant implications for public health. This specific microorganism possesses a wide array of pathogenic factors that enable it to adhere to, colonize, invade, and infect the host. The objective of the current study was to assess the prevalence of *S. aureus*, determine antimicrobial resistance patterns, and identify virulence genes of methicillin-resistant *S. aureus* (MRSA) strains responsible for subclinical mastitis in bovines. A total of 249 milk samples were collected from various farms in the district of Faisalabad. The presence of subclinical mastitis was assessed by using the California mastitis test. Positive milk samples (*n* = 100) were then subjected to standard microbiological techniques for isolation and identification of *S. aureus*. Antibiogram analysis was conducted by using the disc diffusion method to assess antimicrobial resistance. For the molecular detection of *S. aureus* and its virulence genes, the polymerase chain reaction (PCR) was performed with species-specific primers. The overall prevalence of *S. aureus* was found to be 40% (40/100), which was confirmed through molecular detection of the *nuc* gene in 40/40 (100%) of samples using PCR. Antimicrobial susceptibility tests indicated the highest susceptibility to vancomycin, sulfamethoxazole/trimethoprim, erythromycin, gentamicin, ciprofloxacin, and chloramphenicol, while the highest resistance rate was observed against tetracycline. Additionally, 30% of samples (12/40) tested positive for methicillin resistance. PCR analysis revealed that 100% of MRSA-tested isolates harbored the *mecA* and *clfA* genes. Furthermore, the MRSA isolates showed the presence of *pvl*, *hla*, *hlb*, *sec*, *icaA*, i*caD*, *icaB*, and *icaC* genes at rates of 92, 75, 67, 42, 42, 75, 8, and 25%, respectively. These findings underscore the need for stricter aseptic control in dairy farms to prevent disease transmission between animals and ensure the production of safe and uncontaminated food for human consumption.

## 1 Introduction

One of the most severe illnesses observed on dairy farms is bovine mastitis (mammary gland inflammation), which is thought to cause a global loss of 19.7–32 billion USD due to reduced milk production and the number of medicine withdrawal times.^1,2^ Mastitis is caused by *Staphylococcus aureus,* one of the well-known pathogens which causes several severe infections in humans and livestock worldwide. S. aureus is the most significant contributor to subclinical and clinical bovine mastitis.^3^ It is the second most typical source of food poisoning in people because it can produce several enterotoxins in milk and dairy products.^4^ Clinical *S. aureus* mastitis can range in severity from mild, which only manifests itself as changes in milk, to peracute gangrenous mastitis, which results in necrosis of the affected mammary quarter, severe systemic symptoms, and sometimes even death of the cow.^5^

*S. aureus* can cause a wide range of clinical and subclinical symptoms and is partially dependent on the production of several virulence factors that work in concert to provide immune system resistance and enable pathogen adaptation to the host immune system. Surface binding proteins, enterotoxins, superantigens, and leukocidins are the most important virulence factors that can be harbored by infectious *S. aureus* isolates, and they all contribute to the establishment of intramammary infection and aid the pathogen in evading the host immune system. Consequently, the variety of virulence factors in *S. aureus* significantly impacts the manifestation of infections in animals.^6,7^

For mastitis to develop, colonization and attachment are essential. The clumping factors (clfA and clfB), fibronectin-binding proteins (fnbA and fnbB), elastin-binding proteins (ebpS), and the collagen-binding protein (cna) all play significant functions in the attachment of *Staphylococcus* spp to host cells as well as in colonization and penetration.^8^ Additionally, the operon ica (ABDC), which is involved in the production of polysaccharide intercellular adhesin, is essential for the formation of biofilms.^9^

Hemolysins are one of the many exoproteins produced by *S. aureus*, and they help the organism to proliferate and to cause disease in its mammalian hosts.^10^ While most strains identified from intramammary infection in bovine express β-hemolysin, which is a sphingomyelinase, α-hemolysin is cytotoxic. Both toxins are known to promote *S. aureus* adherence to mammary gland epithelial cells.^11,12^

*S. aureus* isolated from bovine mastitis has many superantigenic toxin-coding genes, including staphylococcal enterotoxins (SEs). They are pyrogenic toxins that include SEA to SEE, as well as more recently identified toxins such as SEG to SEI, SEK to SET, SE-like toxin J (SElJ), and SElU to SElY. By directly interacting with the major histocompatibility complex class II molecules of antigen-presenting cells and the V regions of T-cell receptors, the superantigenic toxins exhibit high T-cell mutagenic activity without using the typical antigen presentation method.^43^ Although epidemiological research revealed that the majority of bovine mastitis strains included the *sec* gene and suggested that SEC may be involved in the etiology of bovine mastitis.^13^

Another virulence factor that increases the pathogenicity of bacterial strains can also be produced, and its production is connected to the severity of mastitis. For instance, Panton-Valentine leukocidin, a bicomponent pore-forming toxin, has been suggested as a hypervirulent determinant because it participates in leukocyte destruction and tissue necrosis. It is particularly prominent in severe infections.^14^

The identification of antibiotic-resistant bacteria in bovine mastitis infections and the potential for human transmission through the consumption of unpasteurized dairy products are two additional major public health concerns.^44^ β-Lactam antibiotics have a long history of being abused on dairy farms in treating mastitis, which raises severe public health concerns about the emergence of resistant strains and veterinary drugs in milk.^15,16^ Consequently, livestock-associated MRSA is a worldwide hazard to humans and animals. Methicillin resistance is encoded by the *mecA* gene, which enables resistance to all β-lactam antibiotics.^17^

The present research’s objectives were to isolate and identify *S. aureus* in milk samples from mastitis cases and to comprehend the role of virulence genes in connection to infection. The research work carried out antibiogram studies with molecular profiling in addition to the polymerase chain reaction (PCR)-based virulence gene screening of bacterial isolates.

## 2 Results

### 2.1 Prevalence of *S. aureus* Subclinical Bovine Mastitis

The frequency of subclinical mastitis in milk samples obtained via the California mastitis test (CMT) was 100/249 (40%) (Table 1). Furthermore, 40 of 100 (40%) bovine milk samples were positive for *S. aureus*. Gram staining, catalase, and coagulase tests all yielded positive findings for the *S. aureus* isolates. Additionally, *S. aureus* was found to cause hemolysis in the blood medium and produce circular, golden-yellow colonies in mannitol salt media (Table 2).

**Table 1:** Prevalence of Subclinical Mastitis in buffaloes as Measured by CMT

animal species	total number of animals	total number of samples	no of −ve samples	no of +ve samples	positive samples (%)
buffaloes	60	249	149	100	40%

**Table 2:** Results of CMT Screening in the Collected Samples^a^

CMT grades	examined quarters (no)	positive S. aureus isolates (no)	S. aureus isolates (%)
+++	40	20	50
++	20	10	50
+	30	10	33
total	100	40	100

a Percentages were calculated in comparison to the total number of examined samples of each grade.

### 2.2 Molecular Detection of *S. aureus*

**Figure 1:**
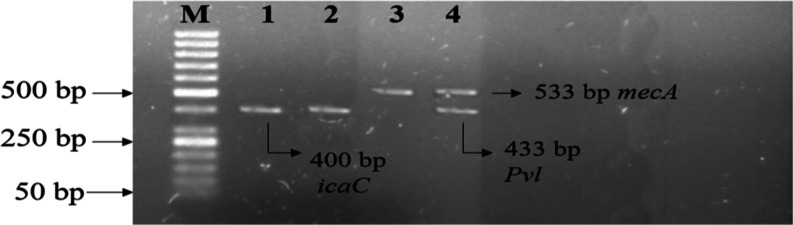
Genotypic characterization of virulence genes of MRSA. Gel electrophoresis analysis of virulence genes of MRSA, ladder size: 50 bp, lanes 1 and 2 represent the positive sample of *icaC* (400bp). Lanes 3 and 4 represent the positive samples of *mecA* (533bp), and lane 4 also represents the positive sample of *pvl* (433).

Furthermore, all strains (*n* = 40) of *S. aureus* exhibiting detectable phenotypes were found to be positive for the *nuc* gene. Consequently, both the phenotypic determination tests and PCR analysis yielded concordant results. The gel electrophoresis image of *nuc* genes is shown in Figure 1.

### 2.3 Antimicrobial Susceptibility Profiles of *S. aureus* Isolates

The antimicrobial susceptibility profile of *S. aureus* isolates demonstrated susceptibility to vancomycin, sulfamethoxazole/trimethoprim, erythromycin, gentamicin, ciprofloxacin, and chloramphenicol. However, this study revealed a notable resistance rate of 30% (12/40) to cefoxitin and 30% (12/40) to oxacillin. It was observed that the isolates resistant to cefoxitin were also resistant to oxacillin, as depicted in (Table 3). Consequently, among the total 40 isolates of *S. aureus*, 12 (30%) demonstrated methicillin resistance, while the remaining 28 (70%) exhibited susceptibility toward methicillin. The phenotypically methicillin-resistant isolates harbored the *mecA* gene; therefore, they were confirmed as MRSA.

**Table 3:** Antimicrobial Susceptibility Pattern of *S. aureus* Isolates

antibiotic	sensitive %	resistance %
vancomycin	(40) 100%	(0) 0%
sulfamethoxazole/trimethoprim	(40) 100%	(0) 0%
erythromycin	(26) 65%	(14) 35%
gentamicin	(40) 100%	(0) 0%
ciprofloxacin	(40) 100%	(0) 0%
chloramphenicol	(40) 100%	(0) 0%
tetracycline	(16) 40%	(24) 60%
cefoxitin	(28) 70%	(12) 30%
oxacillin	(28) 70%	(12) 30%
amoxicillin/clavulanic acid	(40) 100	(0) 0%
ampicillin/sulbactam	(40) 100	(0)% 0

### 2.4 Determination of Virulence Genes of MRSA Strains

**Figure 2:**
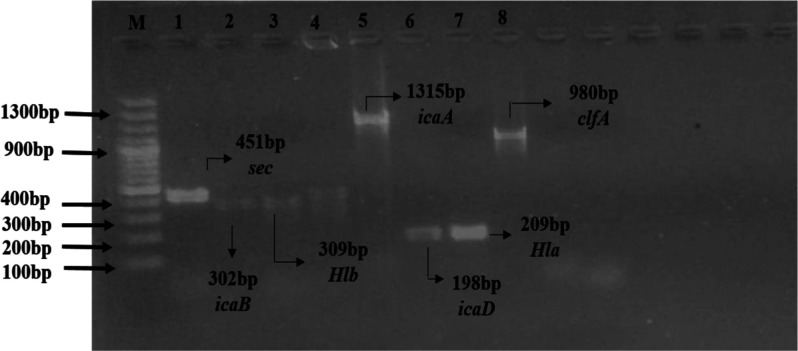
Genotypic characterization of virulence genes of MRSA. Gel electrophoresis analysis of virulence genes of MRSA, ladder size: 100 plus bp, lane 1 represents the positive sample of *sec* (451bp), lane 2 represents the positive sample of *icaB* (302bp), lane 3 represents the positive sample of *Hlb* (309bp), lane 5 represents the positive sample of *icaA* (1351bp), lane 6 represents the positive sample of *icaD*, lane 7 represents the positive sample *Hla* (209bp), and lane 8 represents the positive sample of *clfa* (980bp).

In a molecular analysis of 12 MRSA strains, it was discovered that 5 (42%) of the isolates seemed to have the icaA gene, 1 (8%) had the icaB gene, 3 (25%) carried the icaC gene, and 9 (75%) were positive for the icaD gene. As a result, the highest percentages of icaD and the lowest percentages of icaB were detected in MRSA isolates. Other genes, such as sec, pvl, clfA, Hla, and Hlb, were found in isolates at different percentages. The clfA has a 100% prevalence rate. The prevalence of pvl was observed at 11 (92%). While for hla, it was 9 (75%), for Hlb, it was 8 (67%), and 5 (42%) of the isolates tested positive for the sec gene (Figure 2).

## 3 Discussion

A prominent illness that costs livestock businesses money worldwide is subclinical mastitis. Because of the potential for antibiotic resistance spreading to humans and due to the rise in antibiotic resistance among many bacterial illnesses, the effectiveness of current antibiotic therapy has been negatively impacted, which appears to be an emergent challenge and a serious concern for public health.^18,19^ Additionally, MRSA strains can cause nosocomial infections and have an elevated death rate among humans.^20^ In this study, the frequency of subclinical mastitis was found to be 100/249 (40%). This substantial percentage of subclinical mastitis in bovine is close to the findings from other previous investigations of 44% (88/200).^21^ According to Bangar et al. (2015),^22^ subclinical mastitis affects 46.4% of cattle in India, which is slightly higher than our results. Various diagnostic procedures, sampling techniques, and mastitis measures, as well as aspects, such as the lactation stage, parity number, and breed of animals used in the studies, may contribute to the variance in mastitis prevalence between various studies. Numerous predisposing variables, such as (1) contaminated milking equipment, (2) improper housing, (3) lack of hygiene, and (4) poor animal handling, are implicated in the increased prevalence of subclinical mastitis in dairy livestock. Additionally, there is the failure of treatment, which was always caused by the emergence of bacteria that were resistant to many drugs, a chronic infection that was accompanied by fibrosis, and insufficient dose of antibiotics.^23^

The incidence of *S. aureus* isolated from animals infected with subclinical mastitis was 40% (40 out of 100 CMT-positive milk samples). *S. aureus* was isolated from animals with subclinical mastitis at a rate of 36%,^21^ which is lesser than our results. The biochemical analyses revealed that out of 191 CMT-positive milk samples, 29 (191) had a prevalence of *S. aureus*.^24^*S. aureus* transmission between animals is caused by the use of contaminated milk equipment as well as contaminated milkers’ hands.

In addition, the antimicrobial susceptibility tests showed that 40 isolates were susceptible to vancomycin, sulfamethoxazole/trimethoprim, gentamicin, ciprofloxacin, amoxicillin/clavulanic acid, ampicillin/sulbactam, and chloramphenicol. However, 30% (12/40) of the isolates were resistant to cefoxitin and oxacillin. A previous study showed antibiotic susceptibility, and the isolated *S. aureus* was found to be fully resistant to penicillin, oxacillin, and vancomycin (21%). Contrarily, all isolates were sensitive to tetracycline, gentamycin, norfloxacin, and levofloxacin.^25^ The discovery of clavulanic acid, a naturally existing β-lactamase inhibitor, from *Streptomyces clavuligerus* was a significant step toward the development of novel combinations of the antibiotic. Sulbactam and clavulanic acid (β-lactam antibiotics) have relatively limited bactericidal activity but are powerful β-lactamase inhibitors.

While 65% (26/40) of all isolates were sensitive to erythromycin. Similarly, another study observed 63.1% susceptibility against erythromycin.^26^ Erythromycin is a macrolide antibiotic with a high antibacterial action against Gram-positive and Gram-negative bacteria, such as *staphylococci*, *streptococci*, and *Escherichia coli*.

In the present study (24/40), 60% of bacteria were resistant to the broad-spectrum antibiotic group, such as tetracycline, which has resulted in the emergence of resistant strains due to its widespread use. A previous study revealed that 59.5% of strains were resistant to tetracycline.^27^ Resistance to tetracycline was caused by the ribosomal protection protein produced by*S. aureus*, which competitively binds with tetracycline.

In the current investigation, the antimicrobial profile shows that out of 40 *S. aureus* isolates, 12 (30%) were positive for methicillin resistance, whereas the remaining 28 (70%) were susceptible to methicillin. Out of 85 samples, 25 (29%) samples were MRSA,^28^ and these results were close to our findings. Molecular analysis of the *mecA* gene found that 12 (30%) of the MRSA strains were all positive for it. Another study by Alekish et al. (2020) showed that *mecA* gene was detected in 22.2% strains,^29^ and the prevalence rate was less than in our study. Kaur et al. (2021)^30^ reported a greater prevalence of the *mecA* gene (32.35%), compared to the results of our study. Since the 1990s, a majority of MRSA isolates have had the phenotype of multidrug resistance and harbored several resistant determinants on chromosomes and plasmids. The presence of the chromosomal *mecA* gene of *S. aureus*, which encodes for the production of PBP2a, is ascribed to methicillin resistance. In the presence of β-lactamase enzymes, methicillin remains stable and functional in treating *S. aureus* infection but not against MRSA.^31^

In a molecular analysis of 12 MRSA strains, it was observed that 5 (42%) of the isolates were positive for the *icaA* gene, 1 (8%) had the *icaB* gene, 3 (25%) carried the *icaC* gene, and 9 (75%) were positive for the *icaD* gene. As a result, the highest percentage of *icaD* and the lowest percentage of *icaB* were detected in the MRSA isolates. The *clfA* has a 100% prevalence rate. The prevalence of *pvl* was recorded at 11 (92%). The *pvl* gene is believed to be the most potent staphylococcal leukotoxin capable of resisting neutrophils of bovine. As a result, by destruction of the polymorph-nuclear cells of bovine, pvl may contribute to resistance and boost the pathogenicity against the host. While for *hla*, it was 9 (75%), for *hlb* it was 8 (67%), and 5 (42%) of the isolates tested positive for the *sec* gene. In a study conducted by Roshan et al. (2020),^32^ the presence of virulence factor genes in methicillin-resistant *S. aureus* (MRSA) was investigated. The study reported that the *hlg* gene was detected in 80.9% (34/42) of samples, while the *pvl* gene was found in 47.6% (20/42) of samples. Additionally, the *spa* gene was identified in 92.8% (39/42) of samples.

## 4 Conclusions

Subclinical mastitis caused by *S. aureus* is considered a highly significant public health concern. In the context of bovine milk, this research has shown that the virulence genes most commonly linked to MRSA strains are *clfA*, *pvl*, *hla*, *hlb*, and *icaD*. The current study highlights the importance of *clfA* and *pvl* genes as the most prevalent virulence factors in MRSA strains isolated from bovine milk. Thus, continued research and surveillance of MRSA strains in bovine milk are essential to protecting both animal and human health. By identifying the most prevalent virulence genes and monitoring antimicrobial resistance patterns, stakeholders can develop effective strategies to combat this public health concern.

## 5 Experimental Section

### 5.1 Ethical Consideration

Before starting the research work, ethical approval of the research (GCUF/ERC/18/20) was obtained from the Ethics Review Committee (ERC), Government College University Faisalabad. The samples were collected using techniques per international standard rules for biological samples from bovines.

### 5.2 Sample Collection

A total of 249 row milk samples were collected from different dairy farms in Faisalabad. Animals with subclinical mastitis were selected depending on certain traits, such as (1) showing no obvious signs of the disease and (2) displaying a decrease in milk production, which may result in a high count of somatic cells. Each animal’s udder was palpated before sample collection to look for any physical changes, asymmetry, edema, or other abnormalities. The examiner’s hands, the animal’s udder, and the teats were all cleansed under running water with soap and dried with a fresh towel. To make sure there was no external contamination, 70% ethyl alcohol was used to sanitize the udder, teats, and hands of the tester. The first strips of milk were eliminated and discarded because they might have been tainted by the orifice of the teat. Next, 15–20 mL milk samples were taken from each quadrant and placed in sterilized McCartney bottles with screw-top lids (Thermo Fisher Scientific, Waltham, MA, USA). An ice container was used to transport the milk samples to the lab immediately.^33^

### 5.3 California Mastitis Test

The CMT (screening test) was performed to identify the milk samples that were contaminated with subclinical mastitis. The test procedures were carried out as described earlier. The test relies on the reagent’s interaction with the DNA of somatic cells found in milk.^21^ A small amount of sample of milk was taken from each quadrant of the udder by placing it in a cup with the white plastic paddle along with 2 mL of the scam reagent and gently mixed. Within 20 s, the test findings were assessed visually and classified as (0), (T), +(1), ++(2), and +++(3) depending on the degree of gel formation. Individual milk samples from quarters with high CMT scores were examined bacteriologically. Milk with a CMT score of 1 or more was collected in sterile tubes, transported on ice, and stored at 20 °C until further use.^34,35^

### 5.4 Isolation and Identification of *S. aureus*

The milk samples that were positive for the screening were incubated for 24 h at 37 °C before being centrifuged for 5 min at 3000 rpm. The cream layer was removed, and the sediments were dispersed onto blood agar, nutrition agar, and mannitol salt agar plates. After that, the streaked plates were incubated for 24–48 h at 37 °C. The previously mentioned morphological and biochemical methods were used to identify the presumed growing colonies. For further examination, round convex golden-yellow colonies of *S. aureus* were collected and stored at −80 °C in a medium containing 10% glycerol (v/v).

### 5.5 Antimicrobial Susceptibility Test

The disc diffusion technique was utilized to assess the susceptibility of antibiotics and followed the measures recommended by the Clinical and Laboratory Standards Institute (CLSI).^45,46^ Different antibiotics were employed to determine the susceptibility patterns of the isolates, as outlined in Table 4.

**Table 4:** Interpretive Criteria for Zone of Inhibition Diameter

		diameter of the zone (mm)	
antibiotic	disc conc	resistance	sensitive
vancomycin	30 g	≤14	≥15
sulfamethoxazole/trimethoprim	25 μg	≤10	≥16
erythromycin	15 μg	13 or less	18 or more
gentamicin	10 μg	≤12	≥15
ciprofloxacin	5 g	≤15	≥21
chloramphenicol	30 μg	≤12	≥18
tetracycline	10 g	14 or less	19 or more
cefoxitin	30 μg	≤21 mm	
oxacillin	1 μg	≤10	≥13
amoxicillin/clavulanic acid	10–20 μg	19 or less	20 or more
ampicillin/sulbactam	10 μg	11 or less	15 or more

### 5.6 Detection of MRSA and Its Virulence Genes

PCR was performed for the identification of *S. aureus* and its virulence genes by using species-specific primers for *nuc*, *mecA*, *ica* (*ABCD*), *sec*, *pvl*, *clfA*, *hla*, and *hlb* (Table 5). PCRs were performed in a final volume of 25 μL, which included 12.5 μL of simplex 2× PCR master mix (Green Master, Promega, USA), 5 μL of DNA template, 1 μL of each primer, and 5.5 μL of nuclease-free water. For all PCRs, the initial DNA denaturation step was at 94 °C for 5 min, followed by 30–50 cycles of denaturation for 50 s at 94 °C, and temperatures were set for annealing based on each primer (Table 5), followed by elongation for 1 min at 72 °C, and final extension for 10 min at 72 °C. After PCRs, amplicons were stored at 4 °C until electrophoresis.^36^

**Table 5:** Primer Sequence of Specific Genes with Their Product Size

primer name	sequence	product size	annealing temp	cycles	reference
*icaA*	CCTAACTAACGAAAGGTAG	1315	48°C for 30 s	40	(42)
	AAGATATAGCGATAAGTGC				
*icaC*	TAA CTT TAG GCG CAT ATG TTT	400	54°C for 30 s	50	(38)
	TTC CAG TTA GGC TGG TAT TG				
*icaD*	ATG GTC AAG CCC AGA CAG AG	198	54°C for 30 s	50	(38)
	CGTGTTTTCAACATTTAATGCAA				
*icaB*	CTGATCAAGAATTTAAATCACAAA	302	54°C for 30 s	50	(38)
	AGATGAAGTAGTTGATGTGTATGG				
*sec*	CACACTTTTAGAATCAACCG	451	57°C for 5 s	35	(41)
	CAC ACT TTT AGA ATC AAC CG				
*mecA*	AAA ATC GAT GGT AAA GGT TGG C	533	50°C for 1 min	40	(4)
	ATC TGT ACT GGG TTA ATC				
*nuc*	GCG ATT GAT GGT GAT ACG GTT	270	50°C for min	40	(4)
	AGC CAA GCC TTG ACG AAC TAA AGC				
*pvl*	ATCATTAGGTAAAATGTCTGGACATGATCCA	433	50°C for 45 s	35	(39)
	GCATCAAGTGTATTGGATAGCAAAAGC				
*clfa*	ClfA-F GGC TTC AGT GCT TGT AGG	980	57°C for 60 s	35	(40)
	ClfA-R TTT TCA GGG TCA ATA TAA GC				
Hla	HLA-1CTGATTACTATCCAAGAAATTCGATTG	209	55°C for 30 s	30	(40)
	HLA-2CTTTCCAGCCTACTTTTTTATCAGT				
Hlb	HLB-1 GTGCACTTACTGACAATAGTGC	309	55°C for 30 s	30	(40)
	HLB-2 GTTGATGAGTAGCTACCTTCAGT				

### 5.7 Gel Electrophoresis

PCR products were electrophoresed in 1.5% agarose gel containing ethidium bromide (0.5 μg/mL) in 0.5× TBE (Tris/borate/EDTA) electrophoresis buffer at 90 V for 45 min^37^ using 50 and 1000 bp DNA–ladder (Thermo Scientific, USA). A high-performance UV transilluminator (UV, INC, UK) was then used to see the gel.
